# Clinical Outcomes of Two Versions of the Ahmed Glaucoma Valve FP7 in Glaucoma Patients

**DOI:** 10.7759/cureus.100351

**Published:** 2025-12-29

**Authors:** Styliani Alexia Papadonta, Kalliopi Kontopoulou, Elpida Kollia, Zaira Eleni Armeni, Reem Eliasson, Harald Schilling, Markus Kohlhaas, Sofia Fili

**Affiliations:** 1 Department of Ophthalmology, St. Johannes Hospital, Dortmund, DEU; 2 Department of Ophthalmology, Augenzentrum Dortmund, Dortmund, DEU; 3 Department of Ophthalmology, National and Kapodistrian University of Athens, Athens, GRC; 4 Department of Paediatric Ophthalmology and Adult Strabismus, Moorfields Eye Hospital, London, GBR; 5 Department of Paediatric Ophthalmology, Manchester Royal Eye Hospital, Manchester, GBR; 6 Department of Ophthalmology, Augenklinik-Walsum, Duisburg, DEU

**Keywords:** ahmed glaucoma valve, choroidal detachment, intraocular pressure, ocular hypotony, refractory glaucoma

## Abstract

Background: The primary goal of surgical treatment for glaucoma is the sustained reduction of intraocular pressure (IOP). The Ahmed® Glaucoma Valve (AGV) (New World Medical, Inc., Rancho Cucamonga, CA) is a surgically implanted drainage device that allows bidirectional aqueous humor outflow and IOP regulation. Although multiple generations of the AGV have been developed, comparative studies evaluating patient outcomes remain limited. This retrospective study aimed to compare clinical outcomes following implantation of two different generations of the AGV FP7 in patients with complicated, refractory glaucoma.

Materials and methods: This retrospective study analyzed preoperative and postoperative clinical data from glaucoma patients who underwent AGV FP7 implantation at the Eye Clinic of St. Johannes Hospital in Dortmund, Germany. Two cohorts were compared: group A, treated with an earlier AGV FP7 model between January 2008 and December 2012 (n = 159 eyes), and group B, treated with a newer AGV FP7 model between January 2019 and December 2020 (n = 109 eyes). The newer version features a highly polished surface designed to reduce friction and tissue reaction, whereas the earlier model has a comparatively rougher surface. Clinical outcomes were assessed over a one-year follow-up period and included IOP reduction, number of antiglaucoma medications, changes in visual acuity, postoperative complications, need for reoperation, and overall therapeutic success.

Results: One year postoperatively, IOP reduction was significantly greater in group B (52.5%) compared to group A (42.4%). Despite this difference, the therapeutic target was achieved in both groups. The number of required topical antiglaucoma medications also decreased significantly following AGV implantation by 56.8% in group A and 68.6% in group B. The most common complication at discharge was ocular hypotony, observed in 30.8% of cases in group A and 19.6% in group B. However, no cases of persistent hypotony were reported in either group at the one-year follow-up.

Conclusion: Both the earlier and newer versions of the AGV FP7 effectively provided sustained IOP reduction in patients with refractory glaucoma. Although the newer generation demonstrated a greater IOP reduction and was associated with fewer short-term complications, including hypotony, choroidal detachment, and endothelial touch, overall therapeutic success remained high in both groups.

## Introduction

Glaucoma is a leading cause of irreversible blindness worldwide and represents the second most common cause of blindness in Western Europe, following age-related macular degeneration [1]. In Germany, the prevalence of glaucoma among adults over the age of 40 years is estimated to be between 3% and 5% [2]. Given its substantial impact on vision and quality of life, effective treatment strategies are essential to prevent disease progression. Elevated intraocular pressure (IOP) is the most important modifiable risk factor for glaucoma [3], and both medical and surgical therapies aim to achieve sustained IOP reduction [4-6]. The goal of treatment is to reach a patient-specific target IOP below which further optic nerve damage is unlikely, making IOP reduction the primary metric for evaluating therapeutic success.

Glaucoma drainage devices, which divert aqueous humor through a tube into the deeper orbital tissues, provide a viable alternative to conventional procedures such as trabeculectomy [7]. The Ahmed® Glaucoma Valve (AGV) (New World Medical, Inc., Rancho Cucamonga, CA) is one such implantable drainage system [8]. It consists of a base plate, typically made of medical-grade silicone, polypropylene, or porous polyethylene, a drainage tube, and a valve mechanism composed of medical-grade silicone [1,3,4]. A distinguishing feature of the AGV is its pressure-sensitive valve mechanism, which responds to both elevated and low IOP, thereby reducing the risk of postoperative hypotony [1].

Clinical studies have demonstrated that AGV implantation yields outcomes comparable to those of other surgical treatments [9-17], with some evidence suggesting an advantage in reducing vision-threatening complications [18,19]. Currently, more than 10 AGV models are available, differing in base plate material, size, and target patient populations [3]. This study aimed to compare clinical outcomes and complication rates in glaucoma patients treated with two different versions of the AGV FP7. The newer version features a highly polished surface designed to minimize friction and tissue reaction, while the earlier model has a comparatively rougher surface.

In summary, the primary objective of this study is to compare clinical outcomes and complication rates between patients receiving the older versus newer versions of the AGV FP7, thereby providing clearer guidance on the relative efficacy and safety of these implants.

## Materials and methods

Patients who underwent implantation of an AGV FP7 for complicated, therapy-resistant glaucoma at the Department of Ophthalmology, St. Johannes Hospital, Dortmund, Germany, between January 2008 and December 2012 (group A; n = 159 eyes; older FP7 model) or between January 2019 and December 2020 (group B; n = 109 eyes; newer FP7 model) were included in this retrospective study. The newer version features a highly polished surface, whereas the original model has a comparatively rougher surface.

Patients were eligible for inclusion if they met one or more of the following criteria: (a) presence of medically uncontrolled glaucoma, defined as IOP remaining above target levels despite maximally tolerated medical therapy; (b) history of prior unsuccessful glaucoma surgery or contraindication to trabeculectomy; or (c) advanced glaucomatous optic neuropathy associated with corresponding visual field defects. Exclusion criteria were as follows: (a) active ocular neovascularization; (b) phthisis bulbi or eyes with no light perception; or (c) ongoing ocular inflammation or active uveitis.

Clinical data were retrospectively obtained from hospital records at admission, discharge, and during follow-up visits (one to two weeks, four to eight weeks, three months, and six months), extending up to one year postoperatively. At each visit, assessments included measurement of IOP by Goldmann applanation tonometry, best-corrected visual acuity (BCVA), documentation of the number of antiglaucoma medications (AGMs), and a comprehensive slit-lamp examination. The primary outcome measure was treatment success, evaluated based on IOP, number of required glaucoma medications, BCVA, postoperative complications, and need for additional surgical interventions.

For the purposes of this study, the following definitions were applied: hypotony was defined as an IOP < 6 mmHg on two consecutive examinations [6,19]; if persisting beyond three months postoperatively, it was classified as persistent hypotony. Treatment outcomes were classified as follows: absolute success was defined as maintaining an IOP between 6 and 21 mmHg without the use of AGMs or additional glaucoma procedures; qualified success was defined as maintaining the same IOP range with or without AGMs and/or following additional glaucoma surgery; treatment failure was defined as the occurrence of major complications necessitating valve replacement, explantation, or additional pressure-lowering surgery. These definitions were based on previously published criteria [18,20].

All procedures were performed in accordance with the ethical principles of the latest version of the Declaration of Helsinki and were approved by the institutional ethics committee of St. Johannes Hospital. Due to the retrospective nature of the study, informed consent was waived, as all patient data were anonymized prior to analysis. Quantitative data are presented as mean ± standard deviation (SD). Preoperative and postoperative comparisons were analyzed using paired t-tests, which were appropriate because the same eyes were measured at multiple time points. Normality of differences between paired measurements was confirmed using the Shapiro-Wilk test. Statistical analyses were conducted using SPSS version 26.0 (IBM Corp., Armonk, NY), and a p-value < 0.05 was considered statistically significant. Kaplan-Meier survival curves were used to evaluate treatment success.

AGV implantation at our institution followed a standardized surgical protocol: a conjunctival incision was made at the limbus between two rectus muscles, and a conjunctival pocket was created in the superotemporal or inferonasal quadrant. A sponge soaked with mitomycin C (0.4 mg/mL) was applied and subsequently irrigated with balanced saline solution. The implant was inserted into the conjunctival pocket between the rectus muscles and secured to the sclera with 8-0 polypropylene sutures. The silicone tube was trimmed and beveled to facilitate insertion into the anterior chamber. A 23-gauge needle was used to create a track into the anterior chamber, and the tube was inserted to a depth of approximately 2 mm. The exposed portion of the drainage tube was covered with a Tutopatch® graft, and the conjunctiva was closed with a continuous 10-0 nylon suture.

Postoperative care followed a standardized protocol, including topical steroids and antibiotics. Follow-up visits were scheduled at one to two weeks, four to eight weeks, three months, six months, and 12 months. All surgeries were performed by experienced glaucoma surgeons, and minor variability in surgical technique was minimized by adherence to the standardized protocol.

## Results

Patient demographics, primary glaucoma diagnoses, pre-existing ocular conditions, and previous ocular surgeries for both groups are summarized in Table 1.

**Table 1:** Patient demographics, primary glaucoma diagnoses, pre-existing ocular conditions, and previous ocular surgeries for both groups. YAG: yttrium aluminum garnet.

Patient characteristics	Group A	Group B
Age (years, mean ± SD)	69.7 ± 16.4	69.8 ± 12.8
Gender		
Male	83	50
Female	68	46
Total patients	151	96
Laterality		
Right eye	91	58
Left eye	68	51
Total eyes	159	109
Diagnosis, n (%)		
Primary open-angle glaucoma	68 (42.8)	59 (54.1)
Secondary glaucoma due to uveitis	21 (13.2)	10 (9.2)
Pseudoexfoliation glaucoma	17 (10.7)	25 (22.9)
Secondary glaucoma after multiple surgeries	17 (10.7)	3 (2.8)
Secondary glaucoma due to vascular occlusion	14 (8.8)	1 (0.9)
Secondary glaucoma after trauma	7 (4.4)	2 (1.8)
Juvenile glaucoma	5 (3.1)	1 (0.9)
Aphakic glaucoma	4 (2.5)	2 (1.8)
Pigment dispersion glaucoma	2 (1.3)	3 (2.8)
Secondary glaucoma due to ischemic syndrome	2 (1.3)	1 (0.9)
Angle-closure glaucoma	1 (0.6)	2 (1.8)
Malignant glaucoma	1 (0.6)	0
Pre-existing ocular conditions, n (%)		
Uveitis	28 (17.6)	10 (9.2)
Central retinal vein occlusion	14 (8.8)	0
Amblyopia	14 (8.8)	0
Diabetic retinopathy/diabetic macular edema	10 (6.3)	5 (4.6)
Retinal detachment	9 (5.7)	7 (6.4)
Strabismus	9 (5.7)	0
Nystagmus	8 (5.0)	1 (0.9)
Dry age-related macular degeneration	7 (4.4)	6 (5.5)
Bullous keratopathy/Fuchs endothelial dystrophy	7 (4.4)	3 (2.8)
Epiretinal gliosis	6 (3.8)	13 (11.9)
Band-shaped keratopathy	6 (3.8)	0
Penetrating ocular injury	6 (3.8)	5 (4.6)
Branch retinal vein occlusion	4 (2.5)	3 (2.8)
Contusio bulbi (ocular contusion)	2 (1.3)	0
Central retinal artery occlusion	2 (1.3)	1 (0.9)
Macular hole	2 (1.3)	1 (0.9)
Post-uveitic macular edema	2 (1.3)	2 (1.8)
Wet age-related macular degeneration	1 (0.6)	3 (2.8)
Macular scar	1 (0.6)	0
Nanophthalmos	1 (0.6)	0
Retinochoroidal dystrophy	1 (0.6)	0
Vitreoretinal traction	0	1 (0.9)
Previous ocular surgeries, n (%)		
Trabeculectomy	46 (28.9)	30 (27.5)
Pars plana vitrectomy	35 (22.0)	21 (19.3)
Transscleral cyclophotocoagulation	25 (15.7)	38 (34.9)
Glaucoma surgery (unspecified)	21 (13.2)	7 (6.4)
Exocyclophotocoagulation	18 (11.3)	0
Keratoplasty	16 (10.1)	2 (1.8)
Cyclocryotherapy	10 (6.3)	2 (1.8)
Strabismus surgery	8 (5.0)	1 (0.9)
Deep sclerectomy with mitomycin	4 (2.5)	6 (5.5)
YAG laser iridotomy	4 (2.5)	2 (1.8)
Ex-Press shunt implantation	3 (1.9)	1 (0.9)
Canaloplasty	2 (1.3)	26 (23.9)
Laser trabeculoplasty	1 (0.6)	9 (8.3)
iStent implantation	0	5 (4.6)
XEN implantation	0	4 (3.7)
CyPass implantation	0	5 (4.6)

The progression of mean IOP is presented in Table 2. A comparison of group A and group B was conducted across multiple follow-up time points using independent samples t-tests. At baseline, no significant difference in IOP was observed between group A and group B, confirming comparable preoperative conditions. At discharge, group A demonstrated a significantly lower IOP than group B, reflecting a more favorable immediate postoperative response. During the early follow-up (one to two weeks), IOP values were similar between the groups. However, at four to eight weeks, group A exhibited significantly higher IOP values than group B, suggesting differences in postoperative healing or aqueous outflow regulation during the mid-term recovery period. The difference at three months approached significance but did not reach the conventional threshold (p = 0.052). No significant difference was observed at six months; however, at 12 months, group A showed a renewed elevation in IOP, significantly higher than group B (p = 0.011). Overall, these trends indicate that while group A experiences a more favorable immediate postoperative pressure reduction, its long-term IOP control appears less stable compared to group B.

**Table 2:** Comparison of intraocular pressure between group A and group B across follow-up visits.

Intraocular pressure	Group	n	Mean ± SD	t-value	p-value
Follow-up					
Admission	A	159	28.88 ± 11.01	0.144	0.885
	B	109	28.68 ± 11.36		
Discharge	A	159	8.43 ± 5.05	−2.447	0.015
	B	107	10.07 ± 5.79		
1-2 weeks	A	120	10.38 ± 7.94	−1.292	0.198
	B	79	12.08 ± 10.59		
4-8 weeks	A	115	14.67 ± 9.31	2.026	0.044
	B	90	12.34 ± 6.43		
3 months	A	106	15.71 ± 7.86	1.956	0.052
	B	70	13.6 ± 5.45		
6 months	A	83	14.95 ± 6.28	0.853	0.395
	B	70	14.1 ± 5.97		
12 months	A	75	16.64 ± 7.23	2.591	0.011
	B	55	13.61 ± 5.59		

In both groups, the number of topical AGMs decreased significantly from baseline at all postoperative time points. Table 3 demonstrates the comparison between the two groups. At admission, there was no significant difference between group A and group B, confirming similarity at baseline. At discharge, the difference between groups did not reach statistical significance. Similarly, no significant differences were observed at the one to two-week follow-up or at four to eight weeks. At three months and at six months, the groups remained statistically comparable. By 12 months, group A demonstrated slightly higher scores than group B, but this difference did not reach statistical significance. No significant differences were found between the two groups regarding the reduction in required AGMs.

**Table 3:** Comparison of the number of antiglaucomatous medications between group A and group B across follow-up visits.

Antiglaucomatous medications	Group	n	Mean ± SD	t-value	p-value
Follow-up					
Admission	A	159	2.62 ± 0.96	0.073	0.942
	B	109	2.61 ± 1.27		
Discharge	A	159	0.01 ± 0.16	-1.461	0.145
	B	107	0.07 ± 0.48		
1-2 weeks	A	120	0.14 ± 0.54	-0.119	0.906
	B	79	0.15 ± 0.64		
4-8 weeks	A	115	0.32 ± 0.73	-0.604	0.547
	B	90	0.39 ± 0.93		
3 months	A	106	0.63 ± 1.04	-0.063	0.95
	B	70	0.64 ± 1.02		
6 months	A	83	0.82 ± 1.14	0.108	0.914
	B	70	0.80 ± 1.14		
12 months	A	75	1.13 ± 1.10	1.533	0.128
	B	55	0.82 ± 1.19		

As shown in Table 4, a consistent and clinically relevant difference in BCVA was observed between the two groups throughout the follow-up period. At all postoperative time points, group A exhibited significantly worse BCVA compared to group B. These differences were statistically highly significant across every examination interval. Importantly, this disparity was already present at baseline, indicating that the poorer visual acuity in group A reflects pre-existing conditions rather than an effect of the surgical intervention. Consequently, the postoperative differences in BCVA should be interpreted in the context of these initial baseline imbalances. No statistically significant changes in BCVA were observed within either group when comparing baseline values to all postoperative follow-up visits.

**Table 4:** Comparison of best-corrected visual acuity between group A and group B across follow-up visits.

Best-corrected visual acuity	Group	n	Mean ± SD	t-value	p-value
Follow up					
Admission	A	159	0.17 ± 0.33	-3.26	0.0013
	B	109	0.29 ± 0.27		
Discharge	A	159	0.07 ± 0.13	-2.58	0.01056
	B	107	0.11 ± 0.12		
1-2 weeks	A	120	0.09 ± 0.12	-4.58	<0.001
	B	79	0.20 ± 0.19		
4-8 weeks	A	115	0.12 ± 0.17	-4.35	<0.001
	B	90	0.25 ± 0.24		
3 months	A	106	0.16 ± 0.19	-2.54	0.013
	B	70	0.29 ± 0.40		
6 months	A	83	0.16 ± 0.19	-4.70	<0.001
	B	70	0.35 ± 0.29		
12 months	A	75	0.15 ± 0.21	-4.35	<0.001
	B	55	0.35 ± 0.29		

The most common early postoperative complication in both groups was ocular hypotony (30.8% in group A and 19.6% in group B), followed by microhyphema and macrohyphema (Table 5).

**Table 5:** Early postoperative complication in both groups.

Early postoperative complications	Group A		Group B	
	n	%	n	%
Hypotony (new onset)	49	30.82	21	19.63
Microhyphema	21	13.21	15	13.76
Macrohyphema	16	10.6	11	10.9
Choroidal detachment	12	7.55	3	2.75
Fibrinous inflammatory reaction	6	3.77	0	0.00
Intraocular pressure decompensation	2	1.26	4	3.67
Corneal edema	2	1.26	0	0.00
Elevated intraocular pressure	1	0.63	1	0.92
Subchoroidal hemorrhage	1	0.63	2	1.83
Endothelial touch without corneal decompensation	1	0.63	0	0.00
Conjunctival dehiscence	0	0.00	2	1.83

**Figure 1:**
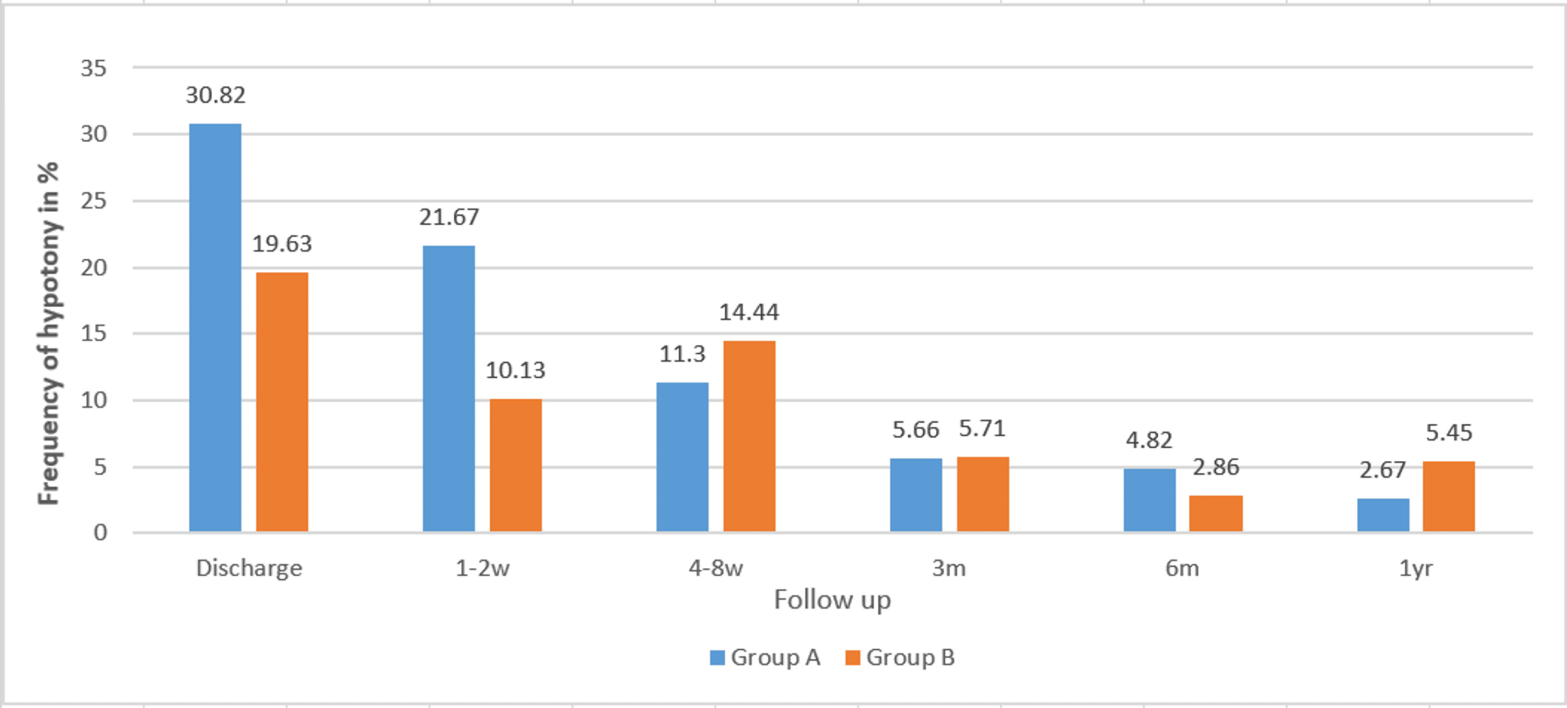
Relative frequency of hypotony as a complication between groups at the time of discharge and during the follow-up period at three, six, and 12 months.

Hypotony was the most common complication in both groups. At discharge and during the early postoperative period, it occurred more often in group A than in group B (Figure 1). Between four and eight weeks postoperatively, hypotony was observed more frequently in group B, while at six and 12 months, the incidence was comparable between both groups.

**Figure 2:**
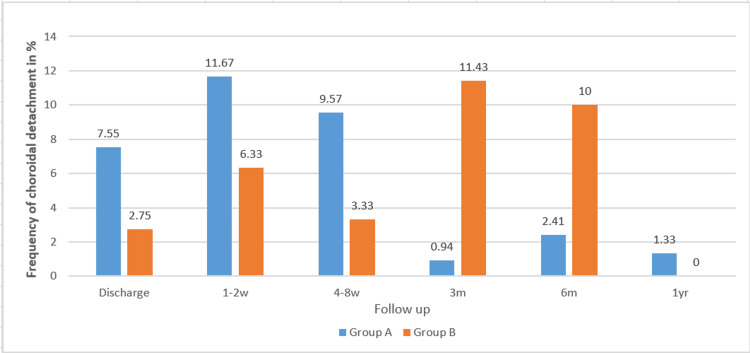
Relative frequency of choroidal detachment as a complication between groups at the time of discharge and during the follow-up period at three, six, and 12 months.

Macrohyphema occurred in 10% of eyes in each group at discharge, but was no longer observed in either group at 12 months. Corneal decompensation, with or without endothelial tube touch, was rare in both groups (n = 9 in group A; n = 15 in group B). Choroidal detachment was more frequent in group A at discharge and shortly thereafter, but was not observed in either group at 12 months postoperatively. Figure 2 illustrates the relative incidence of choroidal detachment at discharge and during follow-up.

Other complications, such as conjunctival dehiscence and cystoid macular edema, were sporadic in both cohorts.

**Figure 3:**
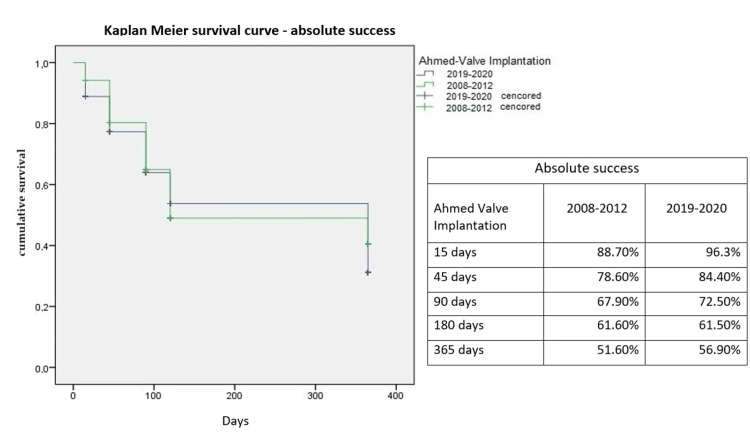
Kaplan-Meier curves for absolute treatment success in both groups.

**Figure 4:**
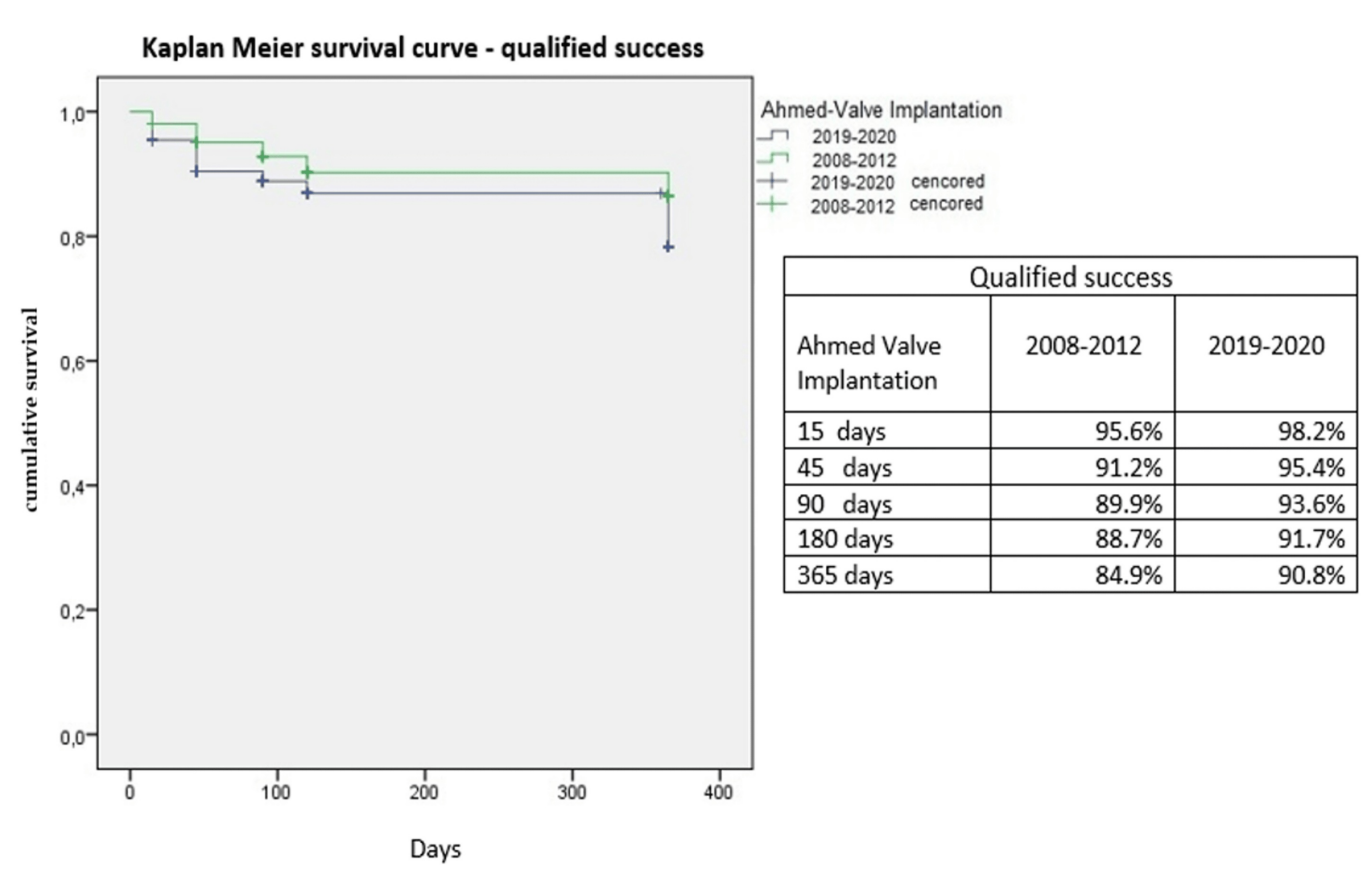
Kaplan-Meier curves for relative treatment success in both groups.

Kaplan-Meier survival curves for treatment success are presented in Figures 3, 4. No statistically significant differences were found between groups in terms of absolute success (p = 0.072) or qualified success (p = 0.14).

Within the first 12 months after AGV implantation, additional pressure-lowering surgery was required in eight of 159 eyes (5%) in group A and 22 of 109 eyes (20.2%) in group B. In both groups, valve replacement due to inadequate pressure control was performed in two cases within four to eight weeks after surgery. In group A, transscleral cyclophotocoagulation was performed in three eyes (1.9%), whereas in group B, this procedure was required in 18 eyes (16.5%) because of persistently elevated IOP. AGV revision was performed in three cases (1.9%) in group A within the first postoperative year; two due to elevated IOP at three months, and one due to IOP elevation associated with encapsulation. In group B, revision surgery was required in two cases (1.8%) within two weeks postoperatively due to IOP decompensation. The frequency of pressure-lowering follow-up surgeries in group A and group B is provided in Table 6.

**Table 6:** Frequency of pressure-lowering follow-up surgeries in group A and group B. AGV: Ahmed® Glaucoma Valve.

Surgeries	AGV revision, n		Transscleral cyclophotocoagulation, n		AGV exchange, n		Number of eyes	
Follow-up	Group A	Group B	Group A	Group B	Group A	Group B	Group A	Group B
Discharge	-	-	-	-	-	-	159	107
1–2 weeks	-	2	-	1	-	-	120	79
4–8 weeks	-	-	-	2	2	2	115	90
3 months	2	-	2	2	-	-	106	70
6 months	1	-	1	6	-	-	83	70
12 months	-	-	-	7	-	-	75	55

Follow-up surgeries due to endothelial touch or corneal decompensation (Table 7) were performed in three eyes (1.9%) in group A; two required tube shortening (one before discharge and one two weeks later), and one underwent penetrating keratoplasty at one year. In group B, 13 eyes (11.9%) required surgical intervention for corneal decompensation or tube-endothelium contact within the first postoperative year. Ten patients (9.2%) underwent tube shortening alone, while in three cases (2.7%), Descemet membrane endothelial keratoplasty (DMEK) combined with tube shortening was performed 12 months after AGV implantation.

**Table 7:** Follow-up surgeries due to endothelial touch or corneal decompensation. DMEK: Descemet membrane endothelial keratoplasty.

Surgeries	Tube shortening without DMEK, n		DMEK plus tube shortening, n		Penetrating keratoplasty, n		Number of eyes	
Follow-up	Group A	Group B	Group A	Group B	Group A	Group B	Group A	Group B
Discharge	1	-	-	-	-	-	159	107
1–2 weeks	1	-	-	-	-	-	120	79
4–8 weeks	-	5	-	-	-	-	115	90
3 months	-	2	-	-	-	-	106	70
6 months	-	2	-	-	-	-	83	70
12 months	-	1	-	3	1	-	75	55

## Discussion

The purpose of this study was to evaluate the treatment outcomes of patients who received different versions of the AGV FP7 implant, as part of the ongoing development of aqueous drainage devices for IOP regulation in glaucoma patients.

The results demonstrate that both versions of the AGV FP7 provided high treatment success rates. A greater IOP reduction was achieved in group B, which received the newer model of the FP7 implant (52.5% reduction), compared to group A (42.4% reduction).

The number of topical antiglaucoma medications was significantly reduced in both groups following AGV implantation; this reduction was 56.8% in group A and 68.6% in group B. The most frequent postoperative complication at discharge in both groups was hypotony; however, no cases of persistent hypotony were observed one year after surgery.

The incidence of choroidal detachment varied between groups depending on the follow-up interval. At discharge and during the first two follow-up visits (one to two weeks and four to eight weeks postoperatively), choroidal detachment was more frequent in group A (older FP7 model). At three and six months, it occurred more often in group B (newer FP7 model), while after one year, it persisted in only one patient from group A. Other complications, such as conjunctival dehiscence and cystoid macular edema, were rare and occurred only sporadically in both groups.

The treatment success rates observed in this study are consistent with previous reports. Budenz et al. [20] reported a cumulative failure probability of 16.4 ± 3.1% one year after AGV implantation in 143 glaucoma patients, which increased to 44.7% after five years [21]. Similarly, Christakis et al. [18] observed a three-year cumulative failure rate of 51% in 124 patients, while Alzendi et al. [22] reported a success rate of 63.1% after a three-year follow-up. In the present study, with a maximum follow-up period of one year, the absolute success rate was 51.6% in group A and 56.9% in group B, while the relative success rate was higher in both groups (84.9% in group A and 90.8% in group B).

Overall, both versions of the AGV FP7 demonstrated comparable efficacy in terms of treatment success, with differences observed mainly in the incidence of postoperative complications. After one year, these complications were rare in both groups, suggesting that neither version of the FP7 implant was superior regarding safety outcomes. These findings are consistent with those of Elbaklish and Gomaa [23], who found no significant difference in treatment success one year postoperatively between two AGV models (S2 and FP7). Likewise, the reduction in medication use compared to baseline was similar in both groups, indicating effective and sustained IOP control with either FP7 version.

When compared with previously published data, the IOP outcomes achieved in the present study can be considered excellent. Budenz et al. [20] reported a mean IOP of 15.4 ± 5.5 mmHg at one year and 14.7 ± 4.4 mmHg at five years postoperatively. Similarly, Elbaklish and Gomaa [23] reported a mean IOP of 16.57 ± 3.35 mmHg at one year.

In the current study, reoperations due to corneal decompensation were infrequent within the first postoperative year. Kim et al. [24] reported a cumulative risk of corneal decompensation of 3.3% five years after AGV implantation, which aligns with the low incidence observed in our cohort.

This study has several strengths, including its clinical focus on refractory glaucoma, a relatively large sample size for a single-center retrospective analysis, and the use of a standardized surgical technique. Outcomes were comprehensively evaluated, including IOP reduction, medication use, visual acuity, postoperative complications, reoperations, and treatment success. Clear definitions for hypotony, absolute and qualified success, and treatment failure were applied, enhancing reproducibility and comparability with previous studies.

The main limitation of this study is its retrospective, single-center design, which limits causal inference and may introduce selection and information biases. Temporal differences between the two study cohorts could have influenced outcomes due to changes in surgical practice or postoperative management. Baseline differences between groups, such as BCVA, may also affect the interpretation of postoperative results. The relatively short one-year follow-up restricts the assessment of long-term safety and efficacy, and the sample size for longer-term outcomes is limited. Additionally, missing or incomplete data inherent to retrospective reviews could have impacted the findings. Consequently, the generalizability of the results to other populations or clinical settings may be limited.

## Conclusions

In summary, both the older and newer versions of the AGV FP7 demonstrated high treatment success in patients with refractory glaucoma. One-year postoperative outcomes showed effective reduction in IOP and antiglaucoma medication use, with no significant differences in efficacy or safety between the two implant versions, indicating comparable and reliable clinical performance.

These findings may guide clinical decision-making when selecting glaucoma drainage devices. Future prospective multicenter studies with longer follow-up are warranted to validate these results and further compare the efficacy and safety of AGV FP7 implants. Such studies could address limitations inherent to retrospective analyses, including selection bias and temporal changes in surgical practice. Baseline differences between groups, particularly in BCVA, and temporal separation of cohorts are acknowledged as potential confounders, and the results should be interpreted in this context.
